# Single-Cell RNA Sequencing of PBMCs Identified Junction Plakoglobin (JUP) as Stratification Biomarker for Endometriosis

**DOI:** 10.3390/ijms252313071

**Published:** 2024-12-05

**Authors:** Thomas Andrieu, Angelo Duo, Lea Duempelmann, Magdalena Patzak, Flurina Annacarina Maria Saner, Jitka Skrabalova, Cinzia Donato, Peter Nestorov, Michael D. Mueller

**Affiliations:** 1 Endometriosis & Gynaecological Oncology Laboratory (EndoGO), Department for Biomedical Research (DBMR), University of Bern, Murtenstrasse 35, 3008 Bern, Switzerland; lea.duempelmann@unibe.ch (L.D.); magdalena.patzak@outlook.com (M.P.); flurina.saner@insel.ch (F.A.M.S.); jitka.skrabalova@unibe.ch (J.S.); michel.mueller@insel.ch (M.D.M.); 2 Inselspital Universitätsspital Bern, Women’s Hospital-Universitätsklinik für Frauenheilkunde, Friedbühlstrasse 19, 3010 Bern, Switzerland; 3 Scailyte AG, True Precision Medicine Through Single-Cell Science, Lichtstrasse 35, 4056 Basel, Switzerland; duo@scailyte.com (A.D.); donato@scailyte.com (C.D.); nestorov@scailyte.com (P.N.)

**Keywords:** Junctional plakoglobin (JUP), endometriosis, adenomyosis, single-cell RNA-sequencing (scRNA-seq), diagnostic biomarkers, peripheral blood mononuclear cells (PBMCs)

## Abstract

This study aimed to identify unique characteristics in the peripheral blood mononuclear cells (PBMCs) of endometriosis patients and develop a non-invasive early diagnostic tool. Using single-cell RNA sequencing (scRNA-seq), we constructed the first single-cell atlas of PBMCs from endometriosis patients based on 107,964 cells and 25,847 genes. Within CD16^+^ monocytes, we discovered *JUP* as a dysregulated gene. To assess its diagnostic potential, we measured peritoneal fluid (PF) and serum JUP levels in a large cohort of 199 patients including 20 women with ovarian cancer (OC). JUP was barely detectable in PF but was significantly elevated in the serum of patients with endometriosis and OC, with levels 1.33 and 2.34 times higher than controls, respectively. Additionally, JUP was found in conditioned culture media of CD14^+^/CD16^+^ monocytes aligning with our scRNA-seq data. Serum JUP levels correlated with endometriosis severity and endometrioma presence but were unaffected by dysmenorrhea, menstrual cycle, or adenomyosis. When combined with CA125 (cancer antigen 125) JUP enhanced the specificity of endometriosis diagnosis from 89.13% (CA125 measured alone) to 100%. While sensitivity remains a challenge at 19%, our results suggest that JUP’s potential to enhance diagnostic accuracy warrants additional investigation. Furthermore, employing serum JUP as a stratification marker unlocked the potential to identify additional endometriosis-related genes, offering novel insights into disease pathogenesis.

## 1. Introduction

Endometriosis is an estrogen-dependent, progressive, and complex chronic gynecologic disease that affects approximately 10% of women of reproductive age worldwide and is characterized by the growth of endometrial tissue outside the uterus [1,2]. It is the main cause of chronic pelvic pain, dysmenorrhea and menorrhagia [3] and is associated with an increased risk of developing OC later in life [4]. The heterogeneity of the clinical presentation challenges a timely and accurate diagnosis of endometriosis. Symptoms are nonspecific, often overlapping with other gynecologic, urologic, and gastrointestinal conditions, leading to misdiagnosis and trivialization of symptoms. This delays referrals from primary care to specialists, resulting in an average diagnostic delay of 7–12 years [5]. Currently, imaging and/or empirical treatment and complementary laparoscopy are recommended for the diagnosis of endometriosis [6]. To date, no clinical blood-based biomarkers have been validated or implemented in clinical practice [7] and the great need remains for a non-invasive diagnosis to reduce the diagnostic delay of patients. In our study we used CA125 and S100A12 (S100 calcium-binding protein A12) for comparative purposes. CA125 is a commonly used biomarker for ovarian cancer routine monitoring [8] but has limited sensitivity for endometriosis diagnosis [9,10]. High levels of CA125 are associated with endometriosis but fluctuate during the menstrual cycle [11,12] and are influenced by chronic pelvic pain [12]. S100A12, on the other hand, is a calcium-binding protein that plays a prominent role in acute inflammatory processes [13]. S100A12 binds to the extracellular domain of RAGE (receptor for advanced glycation end-products), activating the NFκB (nuclear factor-kappa B) pathway. This leads to the release of pro-inflammatory cytokines (e.g., TNF-α (Tumor necrosis factor alpha) and IL-1β (Interleukin-1 beta)) and the upregulation of adhesion molecules, which contribute to inflammation triggered by immune cells [13]. Moreover, it is highly expressed and secreted by monocytes [14]. Elevated levels of S100A12 are found in various inflammatory, neurodegenerative, metabolic, and neoplastic disorders [13]. S100A12 has been investigated as a potential biomarker for Idiopathic Pulmonary Fibrosis [15], Inflammatory Bowel Disease [16] and Myocardial Infarction [17]. Higher mRNA and protein levels of S100A12 were detected in primary endometrial stromal cells (ESCs) isolated from ectopic endometrium (lesions) of patients with endometriosis compared to ESCs isolated from the endometrium of healthy women [18]. S100A12 serum protein has also shown some promise as a blood biomarker for the non-invasive diagnosis of endometriosis [19].

Single-cell RNA sequencing can reveal the unique gene expression of each cell, allowing exploration of cellular heterogeneity, cell type-specific responses to injury and disease, and the underlying mechanisms of these processes. scRNA-seq applied to tissues with infiltrating immune cells recently increased our understanding of cellular cross-talk in a specific microenvironment. This method is very informative in healthy tissues, such as the endometrium [20,21] but also in the context of diseases, such as cancer [22] and endometriosis [23,24,25,26]. In peripheral blood, scRNA-seq is a powerful approach to studying the diversity of immune cells in both healthy individuals [27] and in response to various diseases, including COVID-19 [28], acute respiratory distress syndrome [29], and sepsis [30]. In addition, and even though the studies are sparse, scRNA-seq-based studies can also decipher the adaptation of the PBMCs in the presence of localized or systemic pathologies as demonstrated in Alzheimer’s disease [31], Kawasaki disease [32] and Type 1 Diabetes [33] among others. Given the systemic nature of endometriosis, characterized by the modulation of inflammatory markers in the blood, we hypothesized that PBMCs from endometriosis patients would exhibit distinct characteristics compared to healthy controls.

Junction plakoglobin (JUP, γ-catenin), encoded by the *HGNC* gene on chromosome 17, is expressed in nearly all tissues, with elevated expression in epithelial cells and monocytes. Primarily localized to the cytoplasm (Human Protein Atlas), JUP is also found in cytoplasmic exosomes and the nucleus [34]. As a major component of adherens junctions and desmosomes, JUP connects neighboring cells, limiting proliferation and deformation [35]. Beyond its role in cell-cell adhesion, JUP influences actin microfilament organization and function, thereby modulating cell motility through regulation of cell-extracellular matrix adhesion [35]. Finally, JUP can interact with the p53 signaling pathway to control cell cycle progression and apoptosis, playing both positive and negative roles in diverse malignancies [35]. So far, two studies identified JUP as a potential plasma biomarker. JUP was found in the secretome of atherosclerotic specimens and in resident macrophages of atherosclerotic plaques [36]. Additionally, JUP was identified as a dysregulated protein in OC [37]. This later finding was further supported by an enzyme-linked immunosorbent assay (ELISA) -based screening of a larger patient cohort, showing JUP, in combination with CA125, as a promising early diagnostic test for OC [37].

In our study, we evaluated the ability of scRNA-seq to identify endometriosis-associated differentially expressed genes (DEGs). We also investigated whether JUP, the protein encoded by the major identified DEG, is secreted by immune cells and detectable in the serum, and whether it could be used as a valuable biomarker for endometriosis. The diagnostic performance of JUP was examined independently, in comparison to CA125 or S100A12, and in combination with these markers. Finally, a subgroup analysis was performed using serum JUP level as a stratification marker to potentially identify additional PBMC genes affected in endometriosis.

## 2. Results

### 2.1. ScRNA-Seq of PBMCs Revealed JUP to Be Differentially Expressed with Endometriosis 

**Figure 1:**
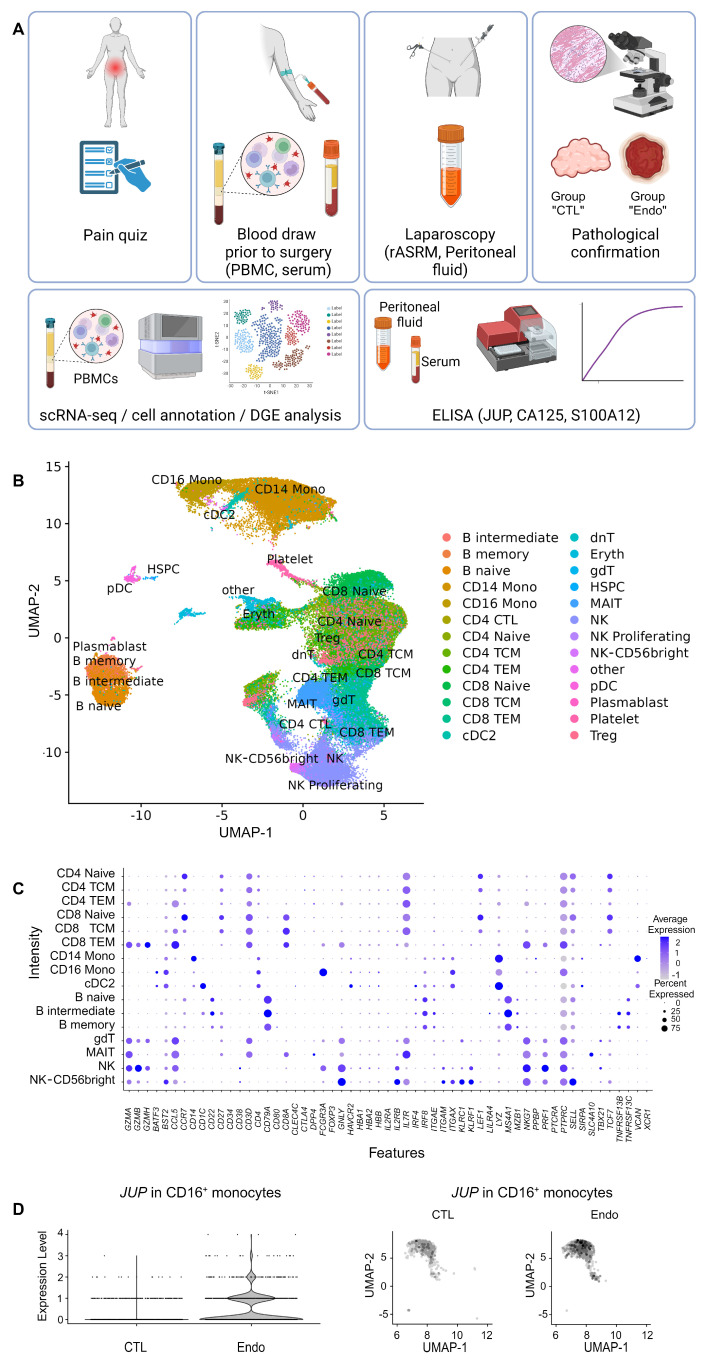
Dysregulated gene expression analysis with endometriosis as endpoint. (**A**) Study design. Pain scores and peripheral blood were collected just before laparoscopy. PBMCs were cryopreserved until single-cell sequencing could be performed by batch. Serums were frozen before quantification of JUP, CA125 and S100A12 by ELISA. During surgery, the revised American Society for Reproductive Medicine (rASRM) score was assigned and suspect lesions were collected for confirmation of diagnosis by a trained pathologist. DGE (differential gene expression) analysis was based on endometriosis diagnosis and level of JUP in serum. (Created in https://BioRender.com, accessed on 8 November 2024) (**B**) Cellular composition of PBMCs based on scRNA-seq (uniform manifold approximation and projection, UMAP plot) for all samples (controls = 7; endometriosis cases = 6). (**C**) Dot plot showing average expression (color scale = expression intensity) and percentage of positive cells (dimension scale = proportion of positive cells) for selected cell type-specific marker genes. (**D**) Comparison of JUP expression profile in endometriosis cases and controls. The left panel represents the expression level of JUP in CD16^+^ monocytes (logFC 1.66; adjusted *p*-value = 0.004). The right panel represents the UMAP of the CD16^+^ monocytes in samples of women without endometriosis (left) and with endometriosis (right). Cells with higher expression levels are indicated by darker dots. Endo: Endometriosis group, CD4 TCM: CD4 central memory, CD4 TEM: CD4 effector memory T cells, CD8 TCM: CD8 central memory, CD8 TEM: CD8 effector memory T cells, cDC2: Type-2 conventional dendritic cells, dnT: TCRα^+^ CD4^−^ CD8^−^ double negative T cells, Eryth: erythrocytes, gdT (Yδt): Gamma-delta (γδ) T cells, HSPC: Hematopoietic Stem and Progenitor Cells, MAIT: Mucosal-Associated Invariant T cells, NK: Natural killer cells, pDC: Plasmacytoid dendritic cells, Treg: regulatory T cells.

ScRNA-seq was performed using PBMCs from women without endometriosis (n = 7) and with endometriosis (n = 6) to identify DEGs associated with endometriosis (Figure 1A).

The demographic characteristics presented in Table S1 (Supplemental Data) showed homogeneous groups regarding surgical findings, inflammatory markers (IL-6 (Interleukin-1 beta), CRP (C-reactive protein), and TNF-α), CA125 expression and cycle phases. Only abdominal pain was slightly higher in the endometriosis group (Mann Whitney test, median of NRS (Numeric Rating Scale for pain) in CTL (control group) = 5.0, in Endo (endometriosis group) = 7.5, *p*-value = 0.03). Mild and severe endometriosis appeared equitably represented but two out of seven rASRM (revised American Society for Reproductive Medicine) scores were missing. The entire cell population was categorized into 21 major cell clusters, from which cell types, including lymphocytes B and T, monocytes, erythrocytes, Natural killer, Treg and plasmablasts, were identified based on the expression of cell type markers (Figure 1B). Key QC (quality control) metrics were within the normal range (Supplemental Table S2 and Figure S1) and the annotation agreed with published cell markers (Figure 1C). No difference in the cell type distribution was observed among the analyzed groups (Supplemental Figure S2). The differential gene expression (DGE) analysis with endometriosis endpoint revealed *JUP* as the only DEG (Supplemental Table S3). *JUP* expression was increased with a log fold change of 1.66 (adjusted *p*-value = 0.004) in CD16^+^ monocytes (Figure 1D). The individual *JUP* expression for each sequenced sample is presented in Supplemental Figure S3.

### 2.2. Serum JUP Is Increased in Endometriosis and OC

**Figure 2:**
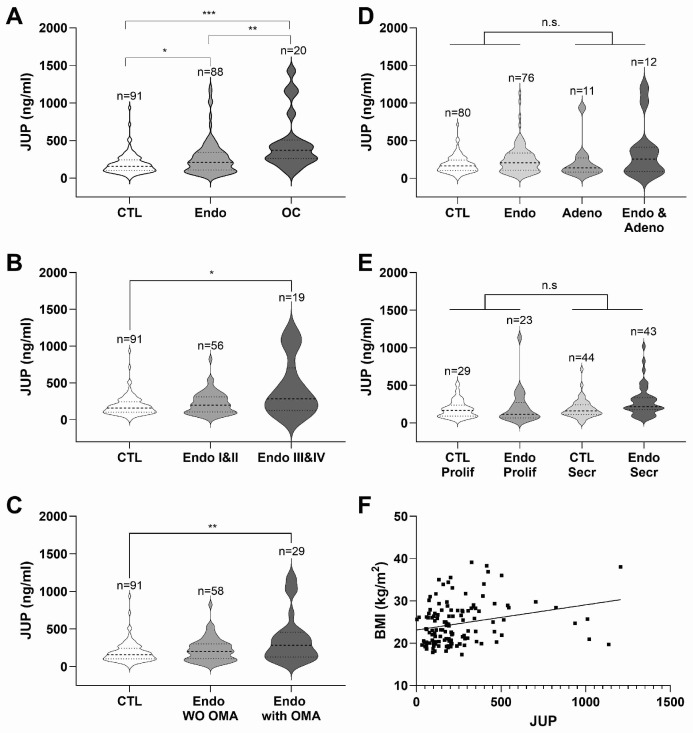
JUP in endometriosis. (**A**) Violin plots of serum JUP level in endometriosis-free women (CTL) (median = 158.5 ng/mL), endometriosis and patients (Endo) (median = 211.4 ng/mL), patients with ovarian cancer (OC) (median = 372.3 ng/mL). CTL vs. Endo (*p*-value = 0.0152), CTL vs. OC (*p*-value < 0.0001), Endo vs. OC (*p*-value = 0.0027) (Mann–Whitney test). (**B**) Violin plots of serum JUP level in CTL (median = 158.5 ng/mL), Endo stage I–II (median = 199.5 ng/mL) and Endo stage III-IV (median = 283.4 ng/mL). Non-parametric one-way ANOVA (analysis of variance, Kruskal–Wallis test, *p*-value = 0.0184) followed by a non-parametric comparison (Dunn’s multiple comparisons test, CTL vs. Endo stage III-IV, *p*-value = 0.019). Patients with adenomyosis were included. (**C**) Violin plots of serum JUP level in CTL (median = 158.5 ng/mL), endometriosis patients without endometrioma (Endo WO OMA) (median = 203.1 ng/mL), and endometriosis patients with endometrioma (Endo with OMA) (median = 283.4 ng/mL). Non-parametric one-way ANOVA (Kruskal-Wallis test, *p*-value = 0.007) followed by a non-parametric comparison (Dunn’s multiple comparisons test, CTL vs. Endo stage III-IV, *p*-value = 0.006). Patients with adenomyosis were included. (**D**) Violin plots of serum JUP level in endometriosis-free women (CTL) (median = 165.5 ng/mL), endometriosis patients (Endo) (median = 207.4 ng/mL), endometriosis-free women with adenomyosis (Adeno) (median = 139.5 ng/mL), and endometriosis patients with adenomyosis (Endo and Adeno) (median = 255.9 ng/mL). Two-way ANOVA assessing the endometriosis and adenomyosis effects (endometriosis, *p*-value = 0.0151; adenomyosis, *p*-value = 0.1167). (**E**) Violin plots of serum JUP level in proliferative phase (CTL Prolif: median = 166.5 ng/mL; Endo Prolif: median = 116.8 ng/mL) and in secretory phase (CTL Secr: median = 162.2 ng/mL and Endo Secr: median = 215.5 ng/mL). Two-way ANOVA assessing the endometriosis and cycle effects (endometriosis, *p*-value = 0.0345; cycle, *p*-value = 0.2251). (**F**) Positive correlation of serum JUP level with BMI (Body Mass Index; Pearson’s r = 0.260, *p*-value = 0.003, and n = 128). (* = *p*-value < 0.05, ** = *p*-value < 0.01, *** = *p*-value < 0.001, n.s. = not significant).

Serum was obtained from 91 women without endometriosis (11 with adenomyosis), 88 with endometriosis (12 with adenomyosis), and 20 with ovarian cancer. The characteristics of the patient cohort can be found in Table S4A–D. JUP serum concentrations were significantly elevated in women with endometriosis (Mann–Whitney test, 1.33 times higher, *p*-value = 0.0152) and OC (2.34 times higher, *p*-value < 0.0001). The level of JUP was significantly higher in OC than in endometriosis (1.76 times higher, *p*-value = 0.0027) (Figure 2A). 

A subgroup analysis was performed to evaluate mean JUP levels according to the endometriosis stage and the presence of endometrioma. Notably, patients with severe endometriosis have higher JUP levels (Figure 2B, one-way ANOVA, *p*-value = 0.0184) and JUP levels were significantly increased in the presence of endometrioma (Figure 2C, one-way ANOVA, *p*-value = 0.0070).

The effects of adenomyosis and menstrual cycle on the JUP level were analyzed independently in the control and endometriosis groups. Patients with unknown menstruation dates and unclear cycle phases were excluded from this analysis (9% and 13% of the control and endometriosis groups, respectively). We found that adenomyosis, which was detected in less than 15% of our cohort, had no effect on the JUP level (two-way ANOVA, *p*-value = 0.1167) (Figure 2D). Similarly, there were no significant differences in JUP level between proliferative and secretory phases in either the control or endometriosis groups (two-way ANOVA, *p*-value = 0.2251) and no interaction (*p*-value = 0.3261) (Figure 2E). In line, JUP was affected neither by the cycle day, nor by the progesterone level (Supplemental Table S5). Even though an association of endometriosis stage with higher dysmenorrhea scores was found in our cohort (Supplemental Table S4C), JUP levels did not correlate with dysmenorrhea, abdominal pain, dyschesia, dysuria, or dyspareunia (Supplemental Table S5). Further correlation analysis did not identify any association between serum JUP levels and patient age or time of blood collection (Supplemental Table S5). Only a weak correlation of serum JUP level with BMI (Body Mass Index) was observed (Pearson’s r < 0.3, *p*-value = 0.003) (Figure 2F, Supplemental Table S5). Due to limited sample size, subgroup analysis of the OC cohort was not feasible. Serum JUP levels for ovarian cancer histotypes, grades, and FIGO (International Federation of Gynecology and Obstetrics) stages are detailed in Table S4D. In summary, the results indicate that JUP is a soluble marker of endometriosis and could be used to identify patients with endometriosis independently of their cycle phase, presence of adenomyosis, pain levels and age.

### 2.3. Combination of JUP and CA125 Identifies Endometriosis with High Specificity

**Figure 3:**
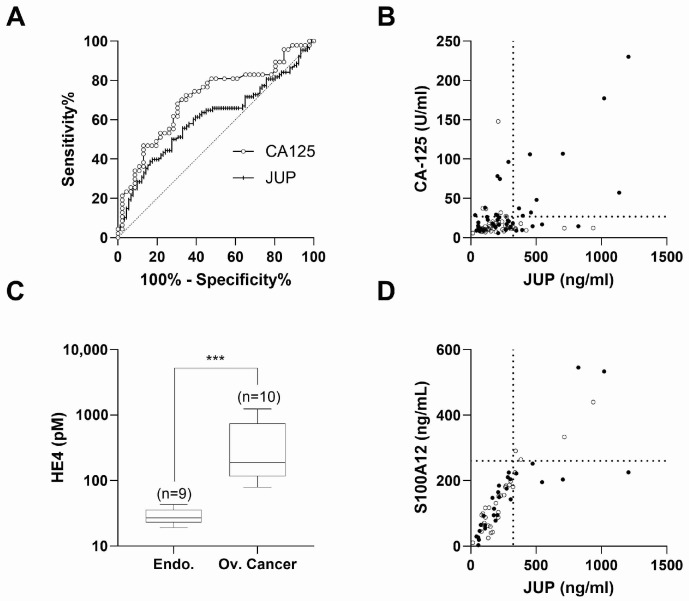
Diagnosis performance of JUP in endometriosis. (**A**) ROC analysis for serum JUP (vertical lines, AUC (Area Under the Curve) 0.6048, *p*-value = 0.0154) and serum CA125 (clear circles, AUC 0.7054, *p*-value = 0.0006). JUP AUC vs. CA125 AUC (*p*-value = 0.1476). (**B**) Positive correlation of JUP with CA125 in serum; Pearson’s r = 0.551; *p*-value 1.020 × 10^−8^; n = 93 (CTL: clear circles; endometriosis: black circles). (**C**) Box and Whiskers plots of HE4 (Human Epididymis Protein 4) quantified in serum in patients with high CA125 (>26.8 U/mL) and high JUP (>324 ng/mL). HE4 was higher in patients with ovarian cancer (median = 189.0 pM) than in patients with endometriosis (median = 26.7 pM) (*p*-value < 0.0001, Mann-Whitney test). (**D**) Positive correlation of JUP with S100A12 in serum; Pearson’s r = 0.835; *p*-value = 2.180 × 10^−16^; n = 59 (CTL: clear circles; endometriosis: black circles). (*** = *p*-value < 0.001).

Next, we calculated the predictive power of JUP alone as a potential in vitro diagnostic marker for endometriosis by constructing a ROC (Receiver Operating Characteristic) curve (Figure 3A). 

With an AUC (Area Under the Curve) of 0.60 (*p*-value = 0.0154, 95% CI (Confidence interval) = 0.5207 to 0.6890), JUP has a significant ability to discriminate cases from controls. At 324 ng/mL, JUP yields a sensitivity of 28% and a specificity of 89% (Supplemental Table S6). CA125 alone showed an AUC of 0.71 (*p*-value = 0.0006, 95% CI = 0.5983 to 0.8124) (Figure 3A) with a predictive sensitivity of 34% and a specificity of 89% at a cut-off of 26.8 U/mL (Supplemental Table S6). The combination of JUP and CA125 decreased sensitivity to 19.15% and increased specificity to 100% (Supplemental Table S6). The relationship between JUP and CA125 is illustrated in Figure 3B (*p*-value < 0.0001, Pearson’s r = 0.551). Clinical data analysis revealed that the JUP/CA125 combination identified at least 35% of severe endometriosis cases, 5% of mild cases, and 40% of patients with endometrioma (Supplemental Table S4E). Additionally, double-positive patients exhibited elevated levels of pro-inflammatory markers (IL-6 and CRP) but similar pain severity compared to negative patients (Supplemental Table S4F,G). CA125 levels, like JUP, can be elevated in both endometriosis and OC. Therefore, we quantified HE4, a known OC biomarker whose level is usually not affected by endometriosis. Interestingly, we found that serums from patients with OC and positive for the combination CA125/JUP all tested positive for HE4 while this was not the case for the endometriosis patients (Figure 3C). The combination of JUP with S100A12 in contrast did not add any benefit to the endometriosis diagnosis compared to JUP. This is in line with the fairly strong correlation existing between both molecules (*p*-value < 0.0001, Pearson’s r = 0.834; Figure 3D). In conclusion, these data show that despite the significantly different JUP values between cases and controls, JUP alone is not a better candidate for a non-invasive biomarker to diagnose endometriosis than CA125, but the combination of JUP with CA125 identifies cases with high confidence. Furthermore, the additional quantification of HE4 makes it possible to distinguish cases of endometriosis from cases of ovarian cancer.

### 2.4. JUP Level Is Low in Peritoneal Fluid and Independent of Endometriosis

Levels of JUP were then assessed in the PF to test whether JUP is secreted into the peritoneal cavity. An ELISA was performed using PF of 130 patients including 57 endometriosis-free women and 73 women with endometriosis at various stages of their menstrual cycle. The ELISA revealed a level of JUP at least 100 times lower in PF than in serum (Supplemental Figure S4A). It is important to note that the mean of JUP in PF was low (8.365 ng/mL ± 17.720 SD), with 41% of the samples being below the detection limit of the assay, which limited the power of analysis. The statistical analysis failed to show any association of PF JUP levels with endometriosis, adenomyosis, cycle phase or dysmenorrhea. (Supplemental Figure S4B–D). Of note, PF JUP values fluctuated with the intensity of dyspareunia (negative correlation, Supplemental Figure S4E). But even when stratifying the patients with dyspareunia, it was not possible to find any strong correlation between PF JUP values and serum JUP values and any association between PF JUP values and endometriosis (Supplemental Figure S4F–H). These results suggested that JUP is not secreted into the peritoneal cavity, but it is a systemic marker of endometriosis.

### 2.5. JUP Is Secreted by Peripheral CD14^+^/CD16^+^ Monocytes

**Figure 4:**
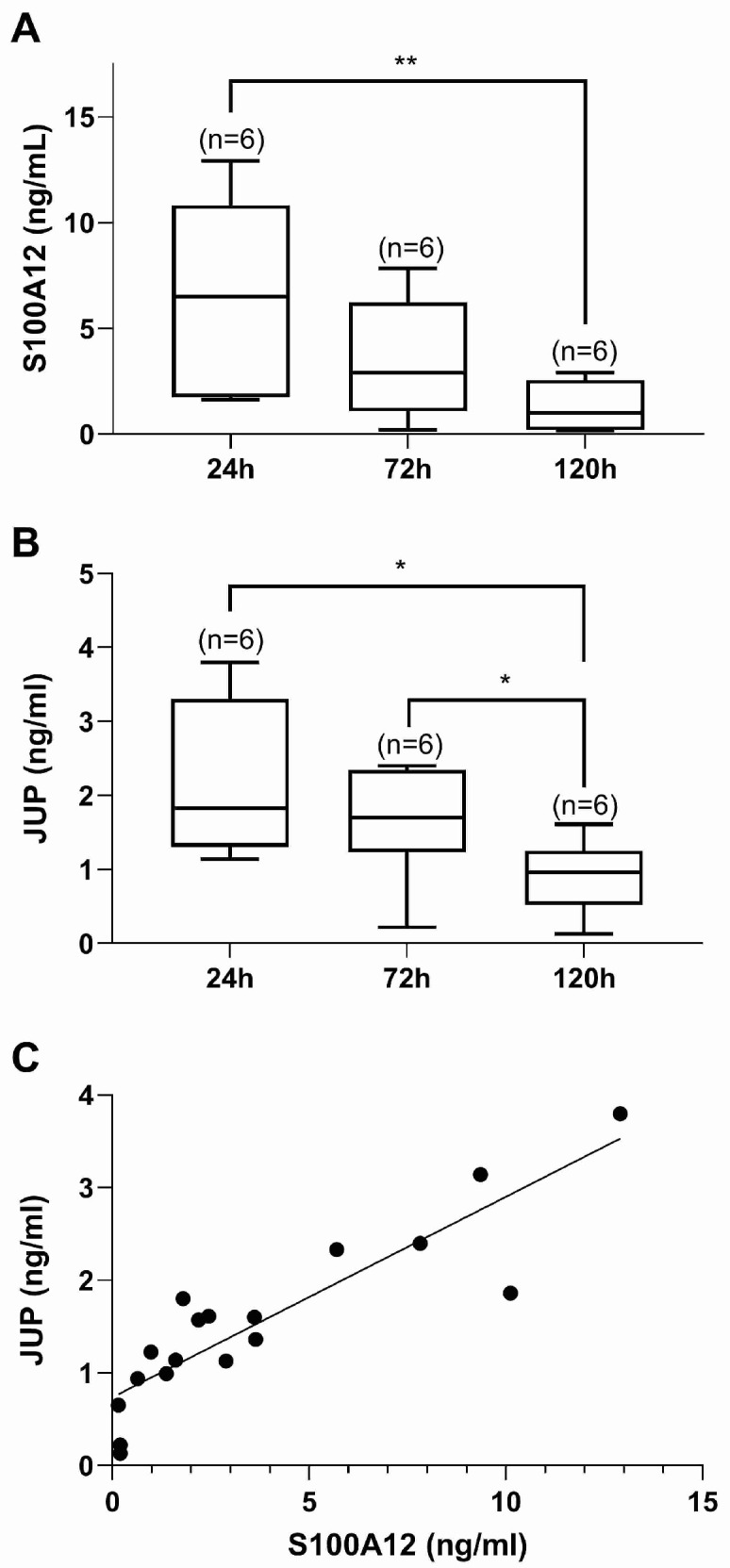
JUP and S100A12 levels in conditioned medium of CD14^+^/CD16^+^ monocytes in culture. (**A**) Box and Whiskers plots of S100A12 quantified in medium at 24 h (median = 6.50 ng/mL), 72 h (median = 2.90 ng/mL), and 120 h (median = 1.00 ng/mL) of in vitro culture. One-way ANOVA (Friedman paired test) (*p*-value = 0.0028). and 24 h vs. 120 h (*p*-value = 0.0073) (Dunn’s multiple comparisons test). (**B**) Box and Whiskers plots of JUP quantified in medium at 24 h (median = 1.83 ng/mL), 72 h (median = 1.70 ng/mL), and 120 h (median = 0.96 ng/mL) of in vitro culture. One-way ANOVA (Friedman paired test) (*p*-value = 0.0054), 24 h vs. 120 h (*p*-value = 0.0424), and 72 h vs. 120 h (*p*-value = 0.0183) (Dunn’s multiple comparisons test). (**C**) Positive correlation of JUP with S100A12 in culture medium; Pearson’s r = 0.889; *p* < 0.0001; n = 18. The values collected at 24 h, 96 h and 120 h for the 6 PBMC cultures were included in this correlation plot. (* = *p*-value < 0.05, ** = *p*-value < 0.01).

JUP expression was significantly increased in peripheral CD16^+^ monocytes of endometriosis patients. Additionally, S100A12 is known to be secreted by CD14^+^/CD16^+^ cells [14]. Considering the strong correlation between JUP and S100A12 in serum (Figure 3D), we hypothesized that the peripheral CD14^+^/CD16^+^ monocytes were able to secrete JUP as well. The monocytes were isolated from peripheral PBMCs using an immunomagnetic negative selection protocol and cultured in the presence of a differentiating factor of up to 5 days. JUP and S100A12 were quantified in the cell supernatant 1, 3, and 5 days after seeding and as previously shown [14], S100A12 secretion decreased with the duration of the culture (Figure 4A). 

Like S100A12, JUP levels decreased during culture (Figure 4B). Notably, a strong correlation between JUP and S100A12 was observed in the CD14^+^/CD16^+^ conditioned medium (Figure 4C) similar to our observation in serum (Figure 3D). Our results therefore suggest that CD14^+^/CD16^+^ monocytes are a source of JUP in the peripheral circulation.

### 2.6. Serum JUP Used as a Stratification Marker Identified Endometriosis-Related Genes in PBMCs

**Figure 5:**
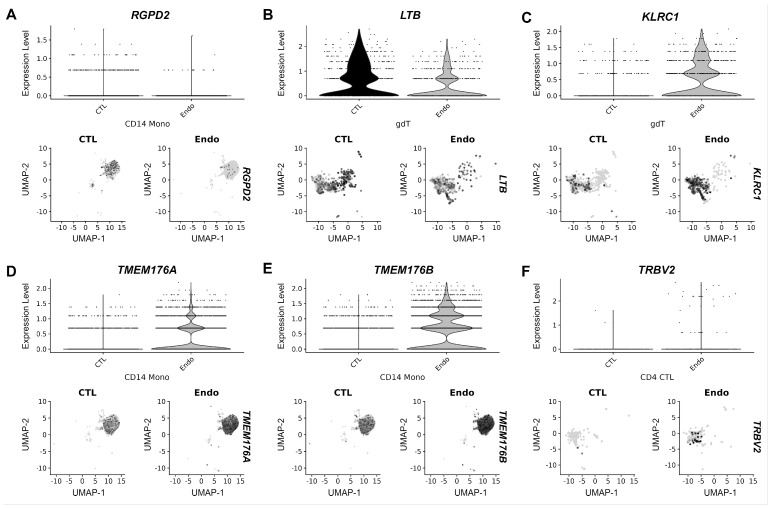
Expression profile of endometriosis DEGs in patients with high (**A**–**C**) and low (**D**–**F**) serum JUP. The upper panels represent the expression level of the DEGs in each identified cluster; (**A**) *RGPD2* in CD14^+^ monocytes; (**B**) *LTB* in γδt; (**C**) *KLRC1* in γδt; (**D**) *TMEM176A* in CD14^+^ monocytes; (**E**) *TMEM176B* in CD14^+^ monocytes; and (**F**) *TRBV2* in CD4 CTLs. The lower panels represent the UMAP of each identified cluster in samples of women without endometriosis (left) and with endometriosis (right). A color code indicates the expression level of the DEGs in each cell; cells with higher expression levels are indicated by darker dots. *KLRC1*: Killer cell lectin-like receptor C1, *LTB*: lymphotoxin beta, *RGPD2*: RANBP2-like and GRIP domain-containing 2, *TMEM176A* and *B*: Transmembrane proteins 176A and B, *TRBV2*: T Cell Receptor Beta Variable 2.

In order to discover additional endometriosis DEGs in PBMCs we further hypothesized that JUP could be used as a stratification marker. To test this hypothesis, we compared DEGs in the function of endometriosis status and of the level of JUP in serum (UMAPs available in Supplementary Figure S5). Comparison between participants without endometriosis (n = 5) and participants with endometriosis (n = 4) with high serum JUP values (>220 ng/mL) identified nine DEGs (*HBB*, *HBA1*, *HBA2*, and *RGPD2* in CD14^+^ monocytes; *CH25H*, *HBB*, *LTB*, and *KLRC1* in γδt; *IFIT2* in NK-CD56bright and *JUN* in Treg). Among these genes, three (*RGPD2*, *LTB*, and *KLRC1*) exhibited distinct expression profiles between control and endometriosis-derived PBMCs (Figure 5A–C). Genes with a less pronounced difference, expressed in a low fraction of cells and at low levels were not depicted. 

All DEGs were down-regulated, except for the up-regulated *KLRC1* (Patient demographics and gene lists are presented in Supplementary Tables S7 and S8). Since, the group of patients with low JUP contained only two participants with endometriosis and 2 participants without endometriosis, additional samples (three controls and four endometriosis cases) have been sequenced and included in the DGE analysis (patient demographics in Supplemental Table S9). This approach identified three DEGs (*TRBV2* in CD4 CTLs; *TMEM176A* and *B* in CD14^+^ monocytes in participants with low serum JUP (<220 ng/mL) (Supplemental Table S8). The expression profile is shown in Figure 5D–F. Our data show that JUP is a novel marker for stratification for endometriosis patients since differences at the transcription level of systemic PBMCs can be revealed based on the level of JUP in serum.

## 3. Discussion

PBMC scRNA-seq data collected from endometriosis cases and disease-free controls represent a first attempt to characterize the cellular diversity and intracellular pathways associated with endometriosis in the peripheral blood. Our study found that elevated *JUP* expression specifically in peripheral CD16^+^ monocytes is a distinctive feature of endometriosis. We did not observe significant alterations in the proportions of different cell types within PBMCs. To the best of our knowledge, this study is the first to identify a link between *JUP* and endometriosis. Using an ELISA test in serum, we have determined that JUP is a new blood biomarker for endometriosis that improves the diagnostic performance of the classic endometriosis marker, CA125. Women with endometriosis had elevated serum JUP levels, which further increased with the severity of the endometriosis and in the presence of endometrioma. Interestingly, JUP levels were independent of dysmenorrhea, menstrual cycle, and adenomyosis. In contrast, JUP was barely detectable in PF, but its secretion was observed in the CD14^+^/CD16^+^ monocyte culture isolated from PBMCs. Stratifying patients based on JUP expression identified additional endometriosis-associated genes that may improve diagnosis, elucidate disease mechanisms, and refine endometriosis subtypes. The stratification analysis suggests indeed a potential alteration in the function or phenotype of CD14^+^/CD16^+^ monocytes, CD4 CTLs, and γδt in endometriosis. Others have also reported dysregulations in PBMCs from women with endometriosis. The number of monocytes in the blood of women with endometriosis is decreased, while their infiltration into inflammatory sites is increased [38]. Circulating T lymphocytes and NK cells also have a lower cytotoxicity in patients with endometriosis [39]. 

JUP improved the diagnostic value of CA125 for endometriosis, mainly by increasing specificity. Blood CA125 has been considered as a potential biomarker in the previous decades, but since elevated serum levels correlate with the severity of endometriosis [40], its use is limited due to its low specificity and sensitivity when considering all types of endometriosis [9,10]. However, our study revealed that JUP is complementary to CA125, as the diagnostic accuracy of CA125 for endometriosis can be enhanced by incorporating JUP, resulting in a specificity of 100% and a sensitivity of 19% in our cohort. This means that a positive test should encourage the clinician to initiate appropriate medical or surgical treatment, according to the “rule-in-test” principle. It is important to note that the JUP validation cohort was significantly enriched with low-grade endometriosis (24 patients with stage I–II and 14 with stage III–IV). This contrasts with our clinic’s general patient population, which typically shows an even distribution of mild and severe endometriosis (25% stage I–II, 25% stage III–IV, and 50% control). This patient distribution, combined with the more pronounced increase in JUP expression in severe endometriosis, likely contributes to the lower identification rate observed in our study cohort. To date, imaging techniques, such as ultrasound (US) and magnetic resonance (MRI), are the most widely recommended methods for diagnosing endometriosis. Pelvic endometriosis can be detected by MRI with a sensitivity of 79% and a specificity of 72%, and transvaginal US has a sensitivity of 65% and a specificity of 95% [41]. Ovarian endometriotic cysts can be diagnosed with transvaginal US with a sensitivity of 93% and a specificity of 96% and with MRI with a sensitivity of 95% and a specificity of 91% [41]. Despite the high overall diagnostic value obtained with US or MRI, these methods have limitations, such as dependence on operator, preparation of the patient, cost, time and a resolution unable to detect the tiny superficial endometriosis lesions. Patient questionnaires are another valuable approach of non-invasive diagnostic tool that is particularly promising for the detection of symptomatic endometriosis [42,43,44,45]. Given the various limitations of the current diagnostic methods, developing simple, non-invasive tests is still essential, and the quest for reliable biomarkers for endometriosis continues to be a highly competitive field of research, with saliva [46] and urine [47] emerging as relevant samples alongside blood. 

The level of JUP in PF was lower than in serum and was not associated with endometriosis. This suggests first that the JUP level is not caused by local tissue damage that might result from ectopic tissue growth and, second, that factors in the peritoneal cavity might inhibit the secretion of JUP. Our results showing that the level of JUP secreted by monocytes decreased with increasing culture time suggest that JUP is predominantly secreted by undifferentiated monocytes. Others found that monocytes purified from PBMCs and cultured in contact with PF collected from women with endometriosis tend to differentiate into macrophages [48]. Therefore, we hypothesize that monocytes from endometriosis patients secrete JUP in serum and lose JUP secretion upon differentiation in contact with PF.

The presence of JUP in serum and conditioned media was rather surprising given that JUP has no signal export (N-terminal signal peptide). There is a small number of important proteins that lack a signal peptide for protein export and do not utilize the ER–Golgi route of protein secretion, such as the signal proteins FGF2 (basic fibroblast growth factor 2), IL-1β, or S100A12 [49,50]. Since JUP and S100A12 levels were strongly correlated in our assays, we speculate that both may share a common release mechanism. It is worth noting that both JUP and S100A12 have previously been detected in extracellular vesicles (http://microvesicles.org/, accessed on 14 March 2023) such as exosomes [51,52]. Whether the detected JUP in endometriosis patients was present as a free protein in serum or packaged in vesicles is not yet known; ELISA tests can indeed detect both forms [49]. Although we observed the presence of JUP in the supernatant of CD14^+^/CD16^+^ monocytes in vitro, we acknowledge the limitation of our approach that does not allow us to confirm that monocytes are the main source of JUP in serum or that monocytes from endometriosis patients secrete more JUP than the control group. 

Single-cell RNAseq in PBMCs identified several DEGs in endometriosis patients after using serum JUP level as a stratification marker. In the group of patients with low JUP levels, we detected upregulated expression of the transmembrane proteins 176A and B. Both *TMEM176A* and *TMEM176B* are normally highly expressed in monocytes, macrophages, and CD11b^+^ dendritic cells. It has been suggested that TMEM176A and TMEM176B inhibit the maturation and activation of dendritic cells in chronic spinal cord injury [53] and that TMEM176B impairs IL-1β secretion and reduces CD8^+^ T cell-dependent antitumor immunity [54]. In the high JUP group, we identified several downregulated genes in the PBMCs from endometriosis patients, including *HBB*, *HBA1*, *HBA2*, *RGPD2*, *CH25H*, *LTB*, *IFIT2* and *JUN*. *KLRC1* was the only upregulated gene detected in this cohort. KLRC1, also known as NKG2A, is involved in the mechanism of self-tolerance that prevents an immune response to self-produced antigens. KLRC1 enables cytotoxic cells to control the expression of major histocompatibility complex class I molecules in healthy cells by recognizing HLA-E (human leukocyte antigen type E). The KLRC1/HLA-E axis plays an important role in modulating the antitumor activity of circulating Vδ2-T cells [55]. Moreover, it has been previously proposed that the KLRC1/HLA-E axis is involved in a hypothetical escape mechanism in which endometriosis ectopic expression of HLA-E inhibits NK cytotoxic activity [56,57,58]. Whether upregulation of KLRC1 affects peripheral Vδ2-T cell activity in women with endometriosis remains to be investigated.

This study has several strengths, including the first scRNA-seq atlas from PBMCs of women suffering from endometriosis and the finding of JUP dysregulation in endometriosis patients. Although the power of this dataset, for the initial discovery of JUP, is limited by the number of samples, a common limitation of scRNA-seq studies given the cost of this technology, it is compensated by validation in a large cohort using an ELISA. The menstrual cycle was also not considered in the performance evaluation of JUP and CA125. Future studies should optimize the performance of CA125 and the JUP/CA125 combination by accounting for menstrual cycle variations in CA125 levels. While an ELISA is suitable for large-scale screening and quantitative analysis, validating JUP and other identified DEGs using reverse transcription polymerase chain reaction or Western blot in enriched cell-type fractions would provide deeper insights into their roles in endometriosis. Furthermore, this study focused on the combination of JUP with CA125 only. A more comprehensive approach, such as stratifying patients based on JUP levels and conducting non-targeted proteomic analysis of blood, could lead to the identification of a more robust panel of biomarkers for a practical and accessible endometriosis diagnostic test. 

Through its numerous interactions, in particular with intracellular proteins (Cadherin 11, RHO A/B/C/H GTPase, beta-catenin, p53) and pathways (Wnt and Vascular endothelial growth factor pathways), JUP was associated with a plethora of roles [35]. Thus, given the results presented here, it is tempting to formulate some hypotheses about the function of JUP in the context of a pathological mechanism. To date, we believe that intracellular JUP may affect monocyte functions, including migration and differentiation, while extracellular JUP represents a marker of extracellular vesicles that may serve as cargo supporting intercellular communication. Further functional assays are required to validate these hypotheses and to find out whether JUP dysregulation in peripheral blood is a cause or consequence of endometriosis.

## 4. Material and Method

### 4.1. Source and Handling of the Biological Material

The workflow is depicted in Figure 1A. Patients undergoing laparoscopy for suspicion of endometriosis, as well as patients without clinical signs and symptoms of endometriosis undergoing laparoscopy for other reasons such as pelvic pain, infertility, chromopertubation, ovarian cysts, sterilization, salpingectomy or hysterectomy, provided informed consent and were requested to complete a pain questionnaire covering information (NRS) on different types of pain including dysmenorrhea, lower abdominal pain, and dyspareunia. Whole blood was drawn before anesthesia for serum preparation. 

PBMCs were obtained from blood (8 mL) collected by venipuncture using Heparin-coated BD vacutainer tubes (CPT tubes, Becton Dickinson, Temse, Belgium, Cat. No. 362780). Blood samples were centrifuged at 1600× *g* for 25 min (acceleration = 7, brake = 1) within two hours of blood collection. Half of the plasma (~4–5 mL) was discarded approximately from each of the CPT tubes. Then, the cell layer was collected and washed according to the provided recommendations. The cells were kept on ice, and CD45-positive cells were counted with CountBright Beads (Thermo Fisher Scientific, Waltham, MA, USA). The cells were cryopreserved in an adequate amount of freezing medium to reach approximately 10 × 10^−6^ CD45^+^/mL. Only samples with viability above 80% after thawing were considered for the sequencing. 

Serum and PF samples were obtained from 179 patients undergoing gynecologic laparoscopy for benign indications. The PF was collected at the beginning of the laparoscopic procedure from the pouch of Douglas. The volume of PF was measured for each patient. Sera and PFs were centrifuged at 1000× g for 10 min and stored in aliquots at −80 °C for subsequent analysis. Serum of women diagnosed with OC was collected at the time of the diagnosis before initial treatment.

### 4.2. Inclusion and Exclusion Criteria

Except for the women with OC, all patients included in this study were operated on by laparoscopy. The indications for laparoscopy included abdominal pain, uterine fibroids, ovarian cysts, or tubal pathologies. The peritoneal cavity of all patients was inspected by experienced gynecologists to detect endometriotic lesions. When lesions were visually detected, a score (stage I–IV) was assigned based on the rASRM score [59] and the subtype (peritoneal, endometrioma, or deep infiltrating) was recorded. All suspicious lesions were removed during laparoscopy and analyzed by an experienced pathologist to validate the endometriotic nature of the biopsy. The total protein content in PF was determined using a micro bicinchoninic acid assay (Quanti-Pro BCA; Sigma-Aldrich, St. Louis, MO, USA) to ascertain the absence of dilution with abdominal flushing medium under the procedure. The PF samples with a total protein content below 15 mg/mL or with hemolysis were excluded from this study. The menstrual phase was assigned based on serum progesterone level and patients’ self-report in which patients were informed about the first day of the last menses (defined as cycle day 1). The proliferative phase has been defined as follows: cycle day below 16 and progesterone level below 1 nmol/L. Progesterone was quantified in the serum by a radioimmunoassay (coat-a-count, DPC; Buhlmann Laboratories, Allschwill, Switzerland) and liquid chromatography high-resolution mass spectrometry (LC-HRMS) [60]. The menstrual phase was not evaluated histologically. Patients were excluded if they were older than 50 years, had acute pelvic inflammation, were treated with hormonal medication (including hormonal contraceptives, intrauterine devices, gonadotropin-releasing hormone antagonists, or progestins) within the past 3 months, were lactating, pregnant, undergoing fertility treatments, or had a history of gynecological infections, malignancies, or recent emergency surgeries. Use of NSAIDs (Nonsteroidal Anti-Inflammatory Drugs) was not reported. 

Patients with OC were undergoing surgery for primary ovarian or fallopian tube cancer at the University Hospital Berne, Switzerland between January 2019 and January 2022. Serum was taken before surgery and all cancer histotypes, grades and FIGO stages were included. Samples of patients younger than 18 years old or who withdrew their consent were excluded.

### 4.3. Single-Cell RNA Sequencing

Cell capture and cDNA library generation were performed using a Chromium system (10x Genomics B.V., Leiden, The Netherlands, Chromium NextGEM Single Cell 5’ Library and Gel Bead Kit v1.1, Cat. No. 1 0001 20 & 1 0001 65), and the cDNA library was sequenced using an Illumina platform. The workflow consisted of steps for quality control of the raw read sequences, transcript quantification, quality control of the samples on a gene- and cell-level, normalization, dimension reduction, and differential gene expression testing. 

The workflow was embedded in the workflow management engine Snakemake [61] for automation and to ensure reproducibility. Cell debarcoding, deduplication, read mapping, and transcript-level expression estimation by pseudo-alignment were performed using the Salmon alevin software [62]. For QC of the raw reads, the software MultiQC [63] was used. QC of the quantification step was performed with the package AlevinQC [62] (https://github.com/csoneson/alevinQC, accessed on 19 February 2021). Seurat [64] v3.1 and Scater [65] were used to perform quality control and visualization of the data on the sample-, cell-, and gene level. Included were the detection and removal of outlier cells based on transcript and gene metrics, detection of possible doublet cells, and batch effects. Cell annotations were obtained using the reference multimodal PBMC dataset in Hao et al. (2021) [66] and the method described by Stuart et al. (2019) [67]. The query resulted in 21 cell types, including lymphocytes B and T, monocytes, erythrocytes, Natural killer (NK), regulatory T cell (Treg), and plasmoblasts. Patient samples were divided into two groups, consisting of those patients who were diagnosed during surgery with endometriosis (Endo) and without endometriosis (CTL). DGE analysis comparing the groups was run using the software package muscat [68] on pseudobulk data (sum of counts per patient and cell type). Genes with an absolute log FC ≥ 1 were retained and considered for further investigation. Similar to above, group comparisons on sample-wise JUP serum levels were made. Using a JUP serum level threshold of 220 ng/mL the samples were stratified into high and low JUP. DGE comparing these groups was performed using muscat on the stratified sample. 

### 4.4. CD14^+^/16^+^ Monocyte Isolation and Culture

PBMCs were thawed in a 37 °C water bath until a few ice crystals remained. PBMCs were transferred in 1 mL of warm (37 °C) RPMI 1640 medium (Life Technologies Europe B.V. Reinach, Switzerland, Cat. No. 11875093) and topped up with 10 mL medium. After centrifugation (300× *g* for 10 min) the supernatant was carefully removed, and the cell pellet was gently resuspended with fresh RPMI 1640 medium. The monocytes were isolated using the EasySep human monocyte enrichment kit without CD16 depletion (StemCell Technologies Inc., Vancouver, BC, Canada, Cat. No. 19058), according to the manufacturer’s instructions. The magnetically labeled cells were separated by using an EasySep magnet (StemCell Technologies Inc., Vancouver, Canada, Cat. No. 18102). The isolated monocytes were cultured in 24-well plates (20 × 10^3^–40 × 10^3^ monocytes/well) in RPMI 1640 medium complemented with 10 mM HEPES (Gibco, Paisley, UK, Cat. No. 15630-56), 2mM L-glutamine (Sigma, St. Louis, MO, USA, Cat. No. A5955), 100 U/mL penicillin, 100 mg/mL streptomycin, (Gibco, Paisley, UK, Cat. No.2503-081) and 10% Fetal Calf Serum (Sigma-Aldrich, St. Louis, MO, USA, Cat. No. F7524) and incubated at 37 °C and 5% CO_2_. They were supplemented with recombinant mouse colony stimulating factor 1 receptor (CSF-1R, 200 ng/mL, BioLegend, San Diego, CA, USA, Cat. No. 792104) to trigger the monocyte-to-macrophage differentiation. On days 1, 3, and 5, the supernatant was collected and replaced with fresh RPMI 1640 medium and CSF-1R. The cell medium supernatant was centrifuged at 3500× *g* for 5 min to remove any cell debris. The samples were concentrated using the Microcon 10kDa Centrifugal Filter (Millipore, Burlington, MA, USA, Cat. No. MRCPRT010) (14,000× *g* for 20 min) and stored at −20°C for further analysis.

### 4.5. Quantification by ELISA 

JUP was quantified in serum and PF using ELISA kits from Anawa (Kloten, Switzerland, Cat. No. MBS2018947). The serum was diluted at 1:250 and the PF at 1:500. S100A12 was quantified in serum using ELISA kits from Cusabio (Houston, TX, USA, Cat. No. CSB-E13095h) with serum dilution of 1:100. CA125 was quantified in serum by ELISA kits from DRG Instruments (Marburg, Germany, TM-CA125 ELISA kit, Cat. No. EIA-5072) with serum dilution of 1:2 in phosphate-buffered saline (PBS). HE4 was quantified using ELISA kits from Fujirebio Diagnostics (Mölndal, Sweden, Cat. No. 404-10) and 25 µL of undiluted serum. All commercially available microplates were performed according to the recommendations of the manufacturers with antigen-antibody incubations at 28 °C in a dry incubator/shaker. All determinations were performed in randomized batches and batch correction was achieved using common samples included in every batch.

### 4.6. Statistical Analysis

The comparisons between the control, endometriosis and OC groups were performed with a non-parametric, unpaired test (Mann-Whitney test). The effects of endometriosis and adenomyosis on serum JUP values were assessed using a 2-way ANOVA. The effect of endometriosis and menstrual effect were assessed using a 2-way ANOVA. The effects of continuous variables (age, BMI, NRS scores, progesterone, CA125, and S100A12) on serum JUP values were assessed by linear regression. The effects of endometrioma and endometriosis grades were assessed independently by non-parametric one-way ANOVA (Kruskal–Wallis test) followed by a non-parametric comparison (Dunn’s multiple comparisons test). The statistical analyses including the ROC curves were performed using GraphPad Prism (version 8.4.3, GraphPad Software Inc. Boston, MA, United States). Values were considered statistically significant at a *p*-value < 0.05. The serum and peritoneal JUP levels were compared using a Wilcoxon paired test.

## 5. Conclusions

Our strategy identified JUP as a novel biomarker for stratification, which in combination with CA125 could be used for highly specific diagnosis of endometriosis. Our results improve our understanding of endometriosis and OC, two diseases with elevated serum JUP levels. Interestingly, using JUP as a stratification marker, additional endometriosis-specific immune dysregulations have been detected, particularly affecting genes of dendritic cell maturation and self-tolerance. More detailed studies with larger data sets and functional studies are needed to accurately determine the role of these dysregulated genes in the pathophysiology of endometriosis.

## Data Availability

The data presented in this study are available for research purposes on request from the corresponding author due to ethical restrictions.

## Supplementary Materials

The following supporting information can be downloaded at: https://www.mdpi.com/article/10.3390/ijms252313071/s1.

## Abbreviations

AUC	Area under the curve
BMI	Body Mass Index
BCA	Bicinchoninic acid assay
CA125	Carbohydrate antigen 125, cancer antigen 125
CD4 TCM	CD4 central memory
CD4 TEM	CD4 effector memory T cells
CD8 TCM	CD8 central memory
CD8 TEM	CD8 effector memory T cells
cDC2	Type-2 conventional dendritic cells
CH25H	Cholesterol 25-hydroxylase
CI	Confidence interval
CTL	Control group
DEGs	Differentially expressed genes
DGE analysis	Differential gene expression analysis
DIE	Deep infiltrated endometriosis
Endo	Endometriosis group
FC	Fold change
FIGO stages	Federation Internationale de Gynecolgie et d’Obstetrique (in French) International Federation of Gynecology and Obstetrics (in English)
HBA1	Hemoglobin subunit alpha 1
HBA2	Hemoglobin subunit alpha 2
HBB	Hemoglobin subunit beta
HLA-E	Human leukocyte antigen type E
IFIT2	Interferon induced protein with tetratricopeptide repeats 2
JUP	Junction plakoglobin
KLRC1	Killer cell lectin-like receptor C1
LC-HRMS	Liquid chromatography high-resolution mass spectrometry
LTB	Lymphotoxin beta
NGS	Next Generation Sequencing
NK	Natural killer
NRS	Numeric Rating Scale for pain
OC	Ovarian cancer
OMA	Endometrioma
PBMCs	Peripheral blood mononuclear cells
PBS	Phosphate Buffered Saline
PCOS	Polycystic ovary syndrome
PF	Peritoneal fluid
Prolif	Proliferative phase
rASRM	Revised American Fertility Society staging system
RGPD2	RANBP2 like and GRIP domain containing 2
S100A12	S100 calcium binding protein A12
scRNA-seq	Single-cell RNA sequencing
SD	Standard deviation
Secr	Secretory phase
SUP	Superficial endometriosis
TMEM176A and B	Transmembrane proteins 176A and B
TNF	Tumor necrosis factor
TRBV2	T Cell Receptor Beta Variable 2
Treg	Regulatory T cell
Yδt	Gamma–delta (γδ) T cells
