# Supplementary material for: Single-Cell RNA Sequencing of PBMCs Identified Junction Plakoglobin (JUP) as Stratification Biomarker for Endometriosis
Source: Int J Mol Sci. 2024 Dec 5;25(23):13071. doi: 10.3390/ijms252313071 (PMC11641628; doi:10.3390/ijms252313071)
Supplement: Supplementary file 1 [file ijms-25-13071-s001.zip › IJMS Supplemental figures V1.3.pdf]

Supplemental figures

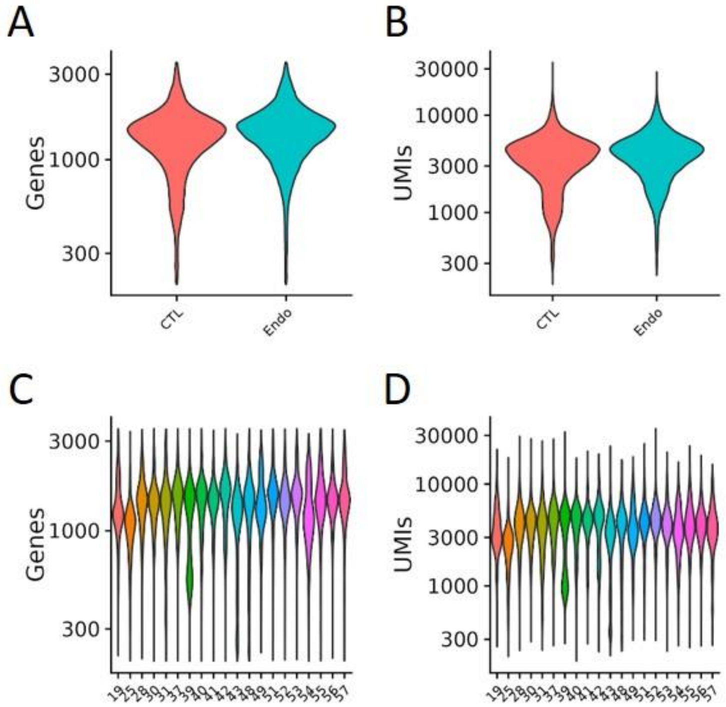

Supplemental Figure S1: Cell-wise number of gene and UMI counts, grouped by disease status **(A, B)** and by sample **(C, D)**.

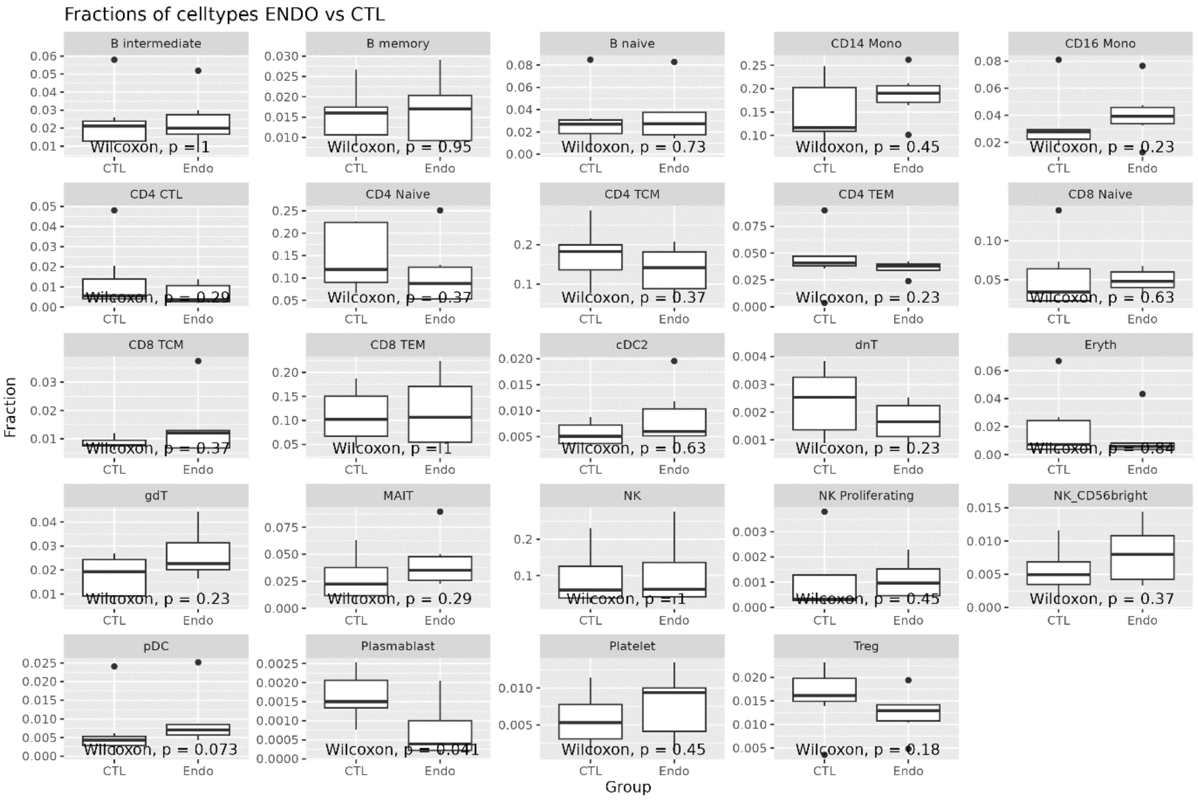

Supplemental Figure S2: Cell counts in endometriosis cases and controls. Unadjusted p values.

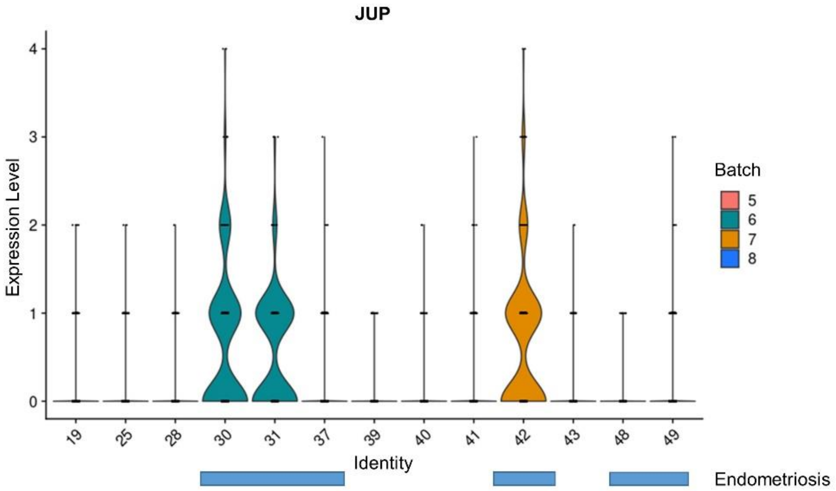

Supplemental Figure S3: Individual JUP expression profile in CD16⁺ monocytes of endometriosis cases and controls. Samples of women with endometriosis were marked with a blue rectangle.

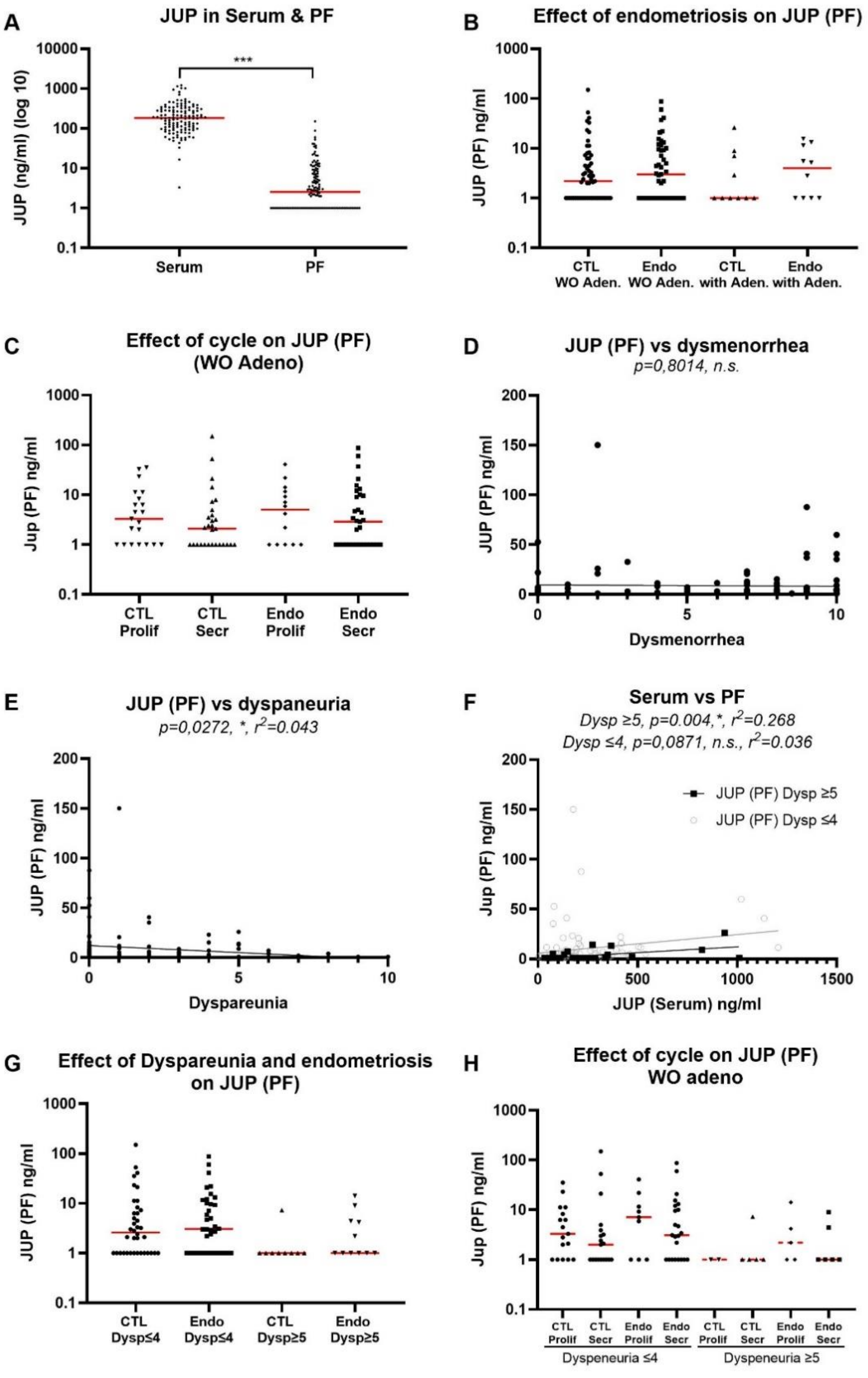

Supplemental Figure S4: Level of JUP in PF in line with the clinical parameters.

(A) JUP level in serum and peritoneal fluid; **(B)** JUP level in PF of women with and without endometriosis or adenomyosis; **(C)** JUP level in PF in proliferative and secretory phases in women with and without endometriosis; **(D)** JUP level in PF in function of dysmenorrhea; **(E)** JUP level in PF in function of dyspaneuria; **(F)** Positive correlation between JUP in PF and serum in women with high dyspaneuria; **(G)** JUP level in PF of women with and without endometriosis stratified with dyspaneuria; **(H)** JUP level in PF of women with and without endometriosis stratified with dyspaneuria and menstrual cycle.

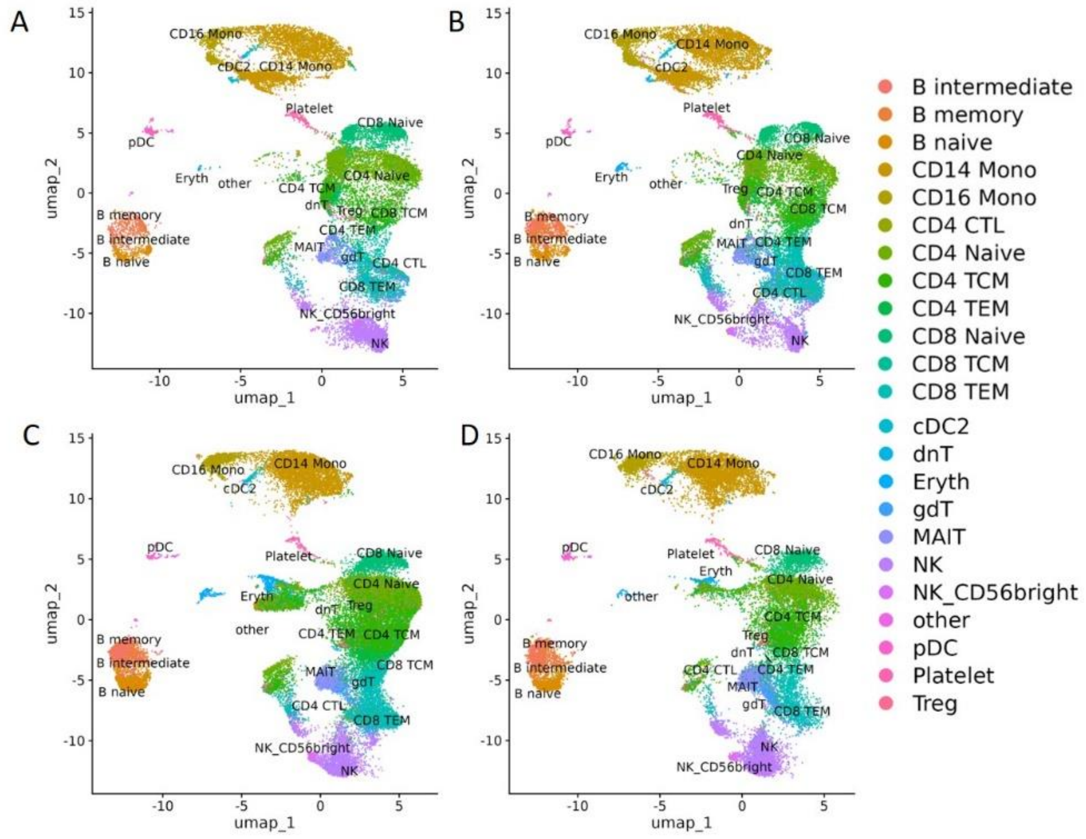

Supplemental Figure S5: UMAP plots for the stratification study.

(A, B) UMAPs in the group with low JUP: **(A)** CTL (n = 5) and **(B)** Endometriosis (n = 6). **(C, D)** UMAPs in the group with high JUP: **(C)** CTL (n = 5) and **(D)** Endometriosis (n = 4).
